# In Vitro Evaluation of 2D-Printed Edible Films for the Buccal Delivery of Diclofenac Sodium

**DOI:** 10.3390/ma11050864

**Published:** 2018-05-22

**Authors:** Georgios K. Eleftheriadis, Paraskevi Kyriaki Monou, Nikolaos Bouropoulos, Dimitrios G. Fatouros

**Affiliations:** 1 Laboratory of Pharmaceutical Technology, School of Pharmacy, Aristotle University of Thessaloniki, 54124 Thessaloniki, Greece; gkelefth@pharm.auth.gr (G.K.E.); paraskemd@pharm.auth.gr (P.K.M.); 2 Department of Materials Science, University of Patras, 26504 Rio, Patras, Greece; 3 Foundation for Research and Technology Hellas, Institute of Chemical Engineering and High Temperature Chemical Processes, 26504 Patras, Greece

**Keywords:** 2D printing, buccal delivery, edible films

## Abstract

Printing technologies have recently emerged in the development of novel drug delivery systems toward personalized medicine, to improve the performance of formulations, existing bioavailability patterns, and patients’ compliance. In the context of two-dimensional printing, this article presents the development of buccal films that are designed to efficiently deliver a class II compound (diclofenac sodium), according to the Biopharmaceutics Classification System (BCS), to the oral cavity. The preparation of drug-loaded inks was carried out based on solubility studies and evaluation of rheological properties, combining ethanol and propylene glycol as optimal solvents. Deposition of the drug was achieved by increasing the number of printing layers onto edible substrates, to produce formulations with dose variance. Thermal analysis, X-ray diffraction, and infrared spectroscopy were used to characterize the developed films. Drug loading and water uptake studies complemented the initial assessment of the films, and preliminary in vitro studies were conducted to further evaluate their performance. The in vitro release profiles were recorded in simulated saliva, presenting the complete release of the incorporated active in a period of 10 min. The effect of multiple layers on the overall performance of films was completed with in vitro permeation studies, revealing the correlation between the number of printed layers and the apparent permeability coefficient.

## 1. Introduction

Printing technologies have emerged in the manufacture of drug delivery systems and dosage forms in the last decade, with a great focus on thermal inkjet printing techniques [1]. The process enables the deposition of liquid droplets containing the active pharmaceutical ingredient (API) onto an edible substrate, while the formulation’s type is based on predesigned digital patterns. In this context, thermal pulses generated by the printing device produce a bubble of steam, the expansion of which forces the liquid ink through a printing nozzle. Thus, a liquid droplet is instantly formed and sprayed onto the substrate [2]. Personalization of one’s dose regarding gender, age, and other genomic features is a promising way to improve public healthcare [3]. Printing technologies are a useful tool in this area, as the adjustment of the dose to the person’s needs is of great interest. In particular, printing techniques are very promising for the fabrication of custom-made oral films as novel drug delivery systems in a tailored, easy, and safe way [4]. The main benefit of this procedure is the personalization of a dose along with the formulation of an API with low solubility in the most common solvents [5].

The rheology of the liquid plays a key role in the two-dimensional (2D) printing procedure. In particular, surface tension and viscosity are the most important parameters to be investigated in order to achieve optimal printability [2,6]. An ink of high viscosity is difficult to be jetted due to possible nozzle clogging, whereas very low viscosities will provoke free-flow of the ink through the nozzle. Aqueous solutions tend to have low viscosity and large surface tension; thus, the use of modifiers is imperative to improve these properties. Polyhydric alcohols and surfactants are commonly used as modifying agents [7]. Moreover, polyhydric alcohols are used as solubility enhancers for poorly soluble drugs. Prednisolone was successfully dissolved in a mixture of water–ethanol–glycerol and printed onto polytetrafluoroethylene (PTFE)-coated fiberglass film [8], and the printability of a folic acid nanosuspension was studied with the aid of Tween 20 [9].

There are numerous studies employing 2D printing, especially inkjet printing. Felodipine, a poorly soluble API and an antihypertensive agent, was ejected onto a hydrophobic substrate made of glass cover slips coated with Flutec fluid. The purpose of this work was to adjust the dosage of the formulations to suit the individual needs of a patient [10]. Buanz et al. [11] highlighted the effect of multiple passes of the substrate through the printer. It was noticed that the amount of salbutamol sulphate deposited on potato starch substrate after a single pass was in agreement with the theoretical loading. Contrariwise, the amount of the API deposited onto the substrate after multiple passes was significantly less than the theoretical dose. The authors proposed that the substrate handling during multiple printing passes caused a large loss of the API, originating from shearing forces. Genina et al. [12] employed 2D printing technologies to deposit loperamide hydrochloride and caffeine on different edible substrates (icing sheets and polyethylene terephthalate sheets). An increase of the concentration of the APIs was achieved by altering the density of the layer which is ejected from the nozzle. The same group [13] formulated orodispersable films by inkjet printing, loaded with rasagiline mesylate, an API against Parkinson’s disease. Three different substrates were used to evaluate the films: copy paper; water-impermeable transparency substrate; and a substrate consisting of HPMC, glycerol, water, and crospovidone. The anticancer drug paclitaxel and the antiviral drug cidofovir were printed in hydroxypropylcellulose (HPC) substrates for the management of cervical cancer, in an attempt to avoid toxicity of the systemic chemotherapy and optimize the treatment [6]. To achieve escalating dosage of APIs, Vakili et al. [14] used prednisolone and levothyroxine in the form of orodispersable films. The printhead ejected the ink solution in a manner of continuous droplets, allowing the uniform deposition of the drug onto the substrate, whereas different doses were printed by adjusting the resolution settings of the device. Flexible doses of levothyroxine have also been developed for pediatric use by modifying the number of printed layers and the resolution, thus attaining lower and higher doses [15]. In another work, Kollamaram et al. [16] used hydroxypropyl methylcellulose (HPMC) as a substrate for printing paracetamol and indomethacin. The study was aimed at the verification of polymorphic selectivity of the APIs when printed, as well as the binding to the substrate and the rheological compatibility of the ink solution. An alternative perspective of printing various doses is the modification of the API’s concentration in the cartridge or changing the dimensions of the predesigned templates. The latter was applied in the case of developing orodispersible films loaded with warfarin [17]. In addition to classical formulation approaches, a recent study presented the perspective of combining inkjet printing with Quick Response (QR) digital technology. The aim of this research was to manufacture smart dosage forms individualized for the person as well as to enable the inclusion of digital information on the printing pattern: expiration date, route of administration, batch number, and manufacturer ID [18].

The aim of the current study is to develop 2D printed oral films for buccal delivery of a BCS class II API [19]: diclofenac sodium (DNa), a nonsteroidal anti-inflammatory drug, commonly used to treat pain and inflammation. Buccal delivery is an alternative route of administration for either local delivery to the oral cavity or systemic release [20]. The buccal route offers the potential of delivering drugs with low bioavailability due to avoidance of the first pass effect [21]. Sugar-sheet substrates are implicated in the manufacture of buccal films. To facilitate the optimization of liquid inks, studies for solubility, viscosity, and surface tension are performed. Buccal films are formulated by altering the number of passes through the printing device to produce various doses of the API. The films are initially assessed for their drug loading and water uptake capacity. Evaluation of the films is complemented by thermal analysis, X-ray diffraction, Raman spectroscopy, and infrared spectroscopy. The in vitro release of the API is performed in simulated saliva. Finally, in vitro permeation studies are used to evaluate the effect of the number of printing passes on the overall performance of the developed films.

## 2. Materials and Methods

### 2.1. Materials

Diclofenac sodium (DNa) was kindly supplied by Rontis Hellas S.A. (Marousi, Greece). Propylene glycol (≥99.5%) (PG), polyethylene glycol 400 (PEG), and ethanol (≥98%) (EtOH) were purchased from Sigma-Aldrich (Steinheim, Germany). Décor Paper Plus edible sugar sheets (A4 dimension) were purchased from Kopyform GmbH (Beindersheim, Germany). All other materials were of analytical grade.

### 2.2. Solubility Studies

Solubility studies of DNa in EtOH and distilled water as the main solvents, as well as in PG and PEG as viscosity and surface tension modifiers, were conducted to optimize the ink composition. In detail, 10 mL of each solvent was placed in airtight glass vials, and preweighted amounts of DNa were gradually added until a cloudy mixture was produced, indicative of exceeding the saturation solubility. The vials were kept under stirring (200 rpm) for 24 h. Aliquots (5 mL) of each mixture were withdrawn, centrifuged at 4000 rcf for 30 min, and filtered through a 0.45 μm Whatman Nylon filter to remove excess amounts of the API, and the supernatant was collected. The saturation solubility of DNa in each solvent was quantified by high-performance liquid chromatography (HPLC).

### 2.3. Development and Printability of Inks

The optimal solvents, providing the highest saturation solubility of the API, were mixed in various ratios to develop drug-loaded inks with appropriate rheological properties for the efficient injection of the ink. Thus, drug-free inks of three different solvent ratios were subjected to kinematic viscosity (ν) measurements in triplicate using a Micro Ostwald viscometer (SI Analytics GmbH, Mainz, Germany). The fluid density (ρ) was measured by weighing 1 mL of each ink in triplicate, to calculate the dynamic viscosity (η) from the equation ν = η/ρ. Furthermore, the viscosity was recalculated for drug-loaded inks, as the addition of API powder would alter the rheology of the liquid formulation. The surface tension of the optimally performing sample was determined by the pendant drop method, using a CAM 200 contact angle goniometer (KSV Instruments, Helsinki, Finland), and the data analysis was performed with the aid of One Attension software (Biolin Scientific, Espoo, Finland).

### 2.4. Printing of Buccal Films

An HP D4260 thermal inkjet printer (Hewlett-Packard Hellas Ltd., Athens, Greece) was involved in the study to prepare the buccal films. The black-ink cartridge was filled with the optimal drug-loaded ink, and square patterns of 2 × 2 cm^2^ were printed on the sugar-sheet substrate. Moreover, the deposition of the drug on the sugar sheets was studied under repeated printing conditions. The substrates were reloaded in the printer five or nine consecutive times to increase the drug content on the square patterns. The buccal films were then carefully extracted using a surgical blade. 

### 2.5. Drug Loading and Water Uptake

The obtained drug-loaded films were dispersed in 50 mL of distilled water in sealed glass vials and kept under stirring (300 rpm) for a period of 4 h at room temperature. Samples of 5 mL were withdrawn and centrifuged at 4000 rcf for 20 min. The supernatant of each sample was collected and analyzed by HPLC to determine the drug loading of the films (expressed as mg/cm^2^). The water absorption capacity was evaluated after immersion of the printed films in Petri dishes containing 1 mL of simulated saliva (SS), pH 6.8 (0.8% sodium chloride, 0.019% potassium phosphate (monobasic), and 0.238% sodium phosphate (dibasic) (*w*/*v*)) [22]. At predetermined time intervals, the films were taken off the Petri dishes, wiped carefully to remove the excess amount of water, and weighed. The water uptake (WU) was calculated as %WU = ((w_2_ − w_1_) × 100)/w_1_)), where w_1_ and w_2_ is the weight of each film before and after immersion in the SS medium.

### 2.6. Physicochemical Characterization

Morphological assessment of the developed formulations was performed with scanning electron microscopy (SEM) using a Zeiss SUPRA 35VP SEM microscope (Zeiss, Oberkochen, Germany). Differential thermal calorimetry (DSC) (5 mg, aluminum pans, 20–350 °C,) of raw materials and drug-loaded films was performed using a DSC 204 F1 Phoenix (Netzsch, Selb, Germany) instrument. Approximately 5 mg of each sample was placed in aluminum pans and the thermograms were recorded in the temperature range of 30–330 °C and a heating rate of 10 °C/min. Additionally, the infrared spectra of the samples (650−4000 cm^−1^, 2 cm^−1^ resolution) were obtained using an IRPrestige-21 (Shimadzu, Kyoto, Japan) instrument. Evaluation of the samples was complemented with X-ray diffraction (XRD) analysis on a Bruker D8-Advance diffractometer (40 kV, 40 mA, Cu Ka1 radiation, 0.35 s/step, Bruker, Billerica, MA, USA), and Fourier transform (FT)-Raman spectra were acquired using a Bruker (D) FRA-106/S component attached to an EQUINOX 55 spectrometer. The excitation source was a R510 diode-pumped Nd:YAG laser at 1064 nm, with a maximum output power of 500 mW.

### 2.7. In Vitro Studies

#### 2.7.1. Drug Release

The in vitro release experiments of DNa-loaded films were performed in SS. Printed films of various doses were enclosed in metal grids, fixed with metal clamps, and immersed in double-walled glass vessels containing 50 mL of SS. The vessels were kept at 37 °C under gentle agitation (100 rpm) for 1 h. Samples of 1 mL were withdrawn at regular time intervals. The samples were centrifuged at 4000 rcf for 20 min, filtered through a 0.45 μm filter, and analyzed by HPLC. The obtained data were analyzed in SigmaPlot v.12.5 (Systat Software, Inc., Chicago, IL, USA) with the aid of a curve fit library (release.jfl). Two nonlinear models (Korsmeyer–Peppas and first-order kinetic) were fitted on the release data, and the equation parameters were assessed.

#### 2.7.2. Permeation Studies

The transport of DNa across a cellulose membrane (Dialysis Tubing, MW cut-off 14000, Sigma-Aldrich, Steinheim, Germany) was studied in Franz diffusion cells (diffusion area 4.9 cm^2^, compartment volume 20 mL). Properly treated cellulose membrane was mounted in the diffusion cells. The acceptor and donor compartments were filled with PBS pH 7.4 and SS medium, respectively. Permeation studies were conducted under constant stirring (110 rpm) at 37 °C. Samples of 1 mL were withdrawn from the acceptor compartment at predetermined time intervals. The samples were centrifuged at 4000 rcf for 20 min, filtered through a 0.45 μm filter, and analyzed by HPLC. The amount of permeated DNa was plotted against time, and the slope of the linear section of the curve determined the steady-state flux (J_ss_). The apparent permeability coefficient (P_app_) was determined as P_app_ = J_ss_/C_d_, where C_d_ indicates the concentration of the active in the donor compartment.

### 2.8. Quantification of DNa

The samples collected from in vitro studies were analyzed in an HPLC system consisting of a LC-10 AD VP pump, a SIL-20A HT autosampler, and a UV–vis SPD-10A VP detector, with a Class VP Chromatography data system v.4.3 interface (Shimadzu, Kyoto, Japan). The stationary phase was a Discovery RP Amide C16 column (15 cm, 4.6 mm, 5 μm). The mobile phase consisted of acetonitrile/KH_2_PO_4_ (0.025 M, pH 3) 50:50 *v*/*v*. The flow rate and injection volume were set at 1 mL/min and 10 μL, respectively. The active compound was detected at 276 nm, with a retention time of 11 min. A linear calibration curve (R^2^ ≥ 0.999) of DNa was observed in the concentration range 1–100 μg/mL.

### 2.9. Statistical Analysis

Values of the executed studies are presented as the mean ± SD. Statistical significance is indicated by *p* < 0.05 values (Student’s *t*-test).

## 3. Results and Discussion

### 3.1. Solubility Studies

The solubility results of DNa in the investigated solvents are presented at Table 1 (values are presented as mean ± SD, *n* = 3). Solubility studies revealed that EtOH increased the solubility of the API more than twofold compared to distilled water, whereas noticeably higher values were obtained compared to its PEG congener. The solubility testing in PG was terminated at a content of 8 g/10 mL. Although at this point, the solvent was capable of dissolving further amounts of the API, handling of the solution was unfavorable due to inappropriate increase in viscosity. However, this solubility potential showed PG to be a suitable viscosity and surface tension modifier of the ink, as well as the overall optimal solvent for DNa.

### 3.2. Viscosity and Surface Tension of the Liquid Ink

Viscosity and surface tension values of inks are of paramount importance for successful printing. Viscosity and surface tension of inks should be within certain limits: 1–30 mPa·s and 25–50 mN·m^−1^, respectively [6,12]. Considering the viscosity increase after addition of the API, various ratios of drug-free solvents were studied and evaluated to select the optimal EtOH/PG ratio for the liquid carrier with a minimum viscosity. Table 2 presents the viscosity values of 20:80, 40:60, and 50:50 (*v*/*v*) ratios of EtOH/PG and the surface tension measurements of the optimal compositions (values are presented as mean ± SD, *n* = 3). A minimum dynamic viscosity of 5.23 mPa·s was calculated in the case of equal volumes of the two solvents. In a similar way, increasing amounts of API were added in the solvent mixture to produce a liquid ink with appropriate viscosity. The overall optimal drug-loaded ink was calculated at 17.64 mPa·s for EtOH/PG 50:50 (*v*/*v*) with a drug content of 227.3 mg/mL, whereas a large content of API (375 mg/mL) produced a liquid ink with viscosity values too close to the upper accepted limit. Surface tension of the drug-free 50:50 (*v*/*v*) sample was measured at 25.7 ± 0.4 mN·m^−1^, increasing to 27. 9± 0.5 mNm^−1^ after addition of the API.

### 3.3. Drug Content and Water Uptake of the Developed Films

**Figure 1:**
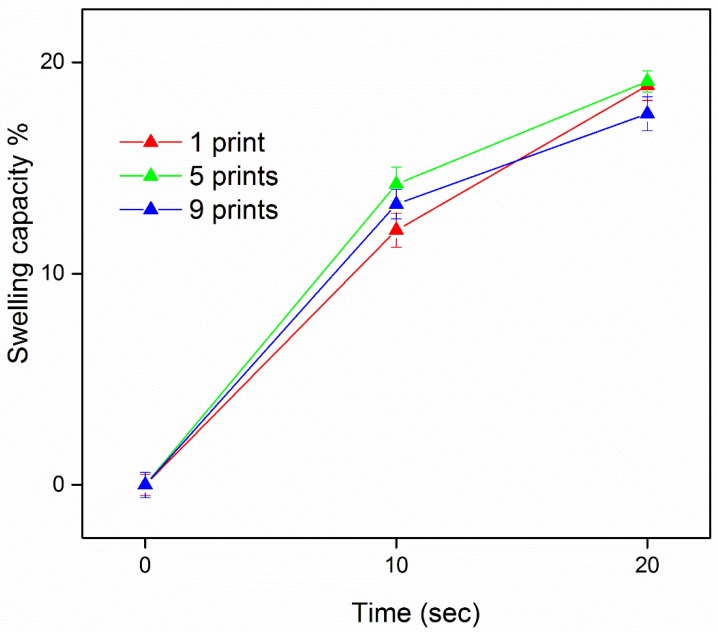
The water uptake capacity of films with a varying number of consecutive prints.

The ink cartridges were filled with the optimal drug-loaded liquid ink to develop buccal films, with deposition of the drug on edible sugar sheets. Increasing doses were produced by varying the number of substrate passes through the printing device. Regarding the printing repetitions, quantification by HPLC presented an increase in the drug content of films. In accordance with previous findings [11], the doses were not proportionally increased, revealing possible drug losses that occur due to the applied shearing forces on the sheets from the printer’s parts during reloading and reprinting. Thus, the DNa content deposited on the developed films was 151 ± 2 μg/cm^2^, 451 ± 4 μg/cm^2^, and 602 ± 4 μg/cm^2^ (mean ± SD, *n* = 3), for 1, 5, and 9 repetitions of printing, respectively. The water uptake results are illustrated in Figure 1 (values are presented as mean ± SD, *n* = 3). The investigation of film capability to absorb SS molecules was feasible for times up to 20 s, as immersion in the medium for a longer time period resulted in deformation of the film structure. Independently of the number of prints (*p* > 0.05), the water absorption capacity for all formulations was in the range of 12–15% at 10 s and 17–19% at 20 s.

### 3.4. Physicochemical Characterization

**Figure 2:**
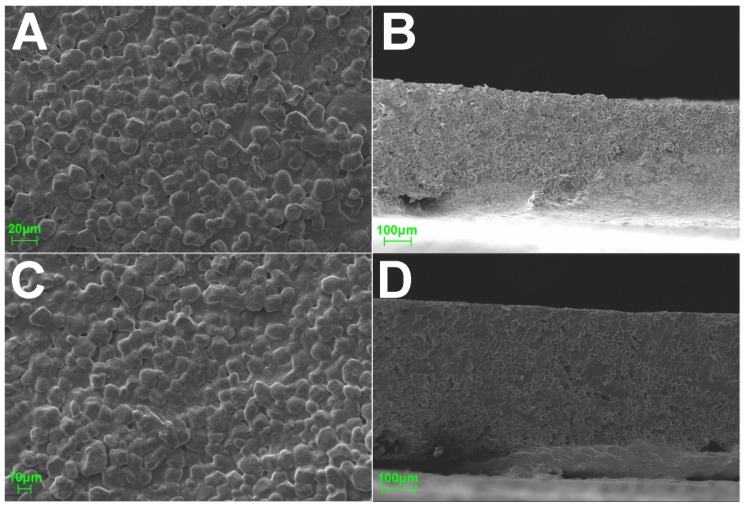
SEM micrographs of the upper and side surfaces of plain (**A**,**B**) and nine-pass (9)-printed (**C**,**D**) sugar sheets.

Figure 2 shows SEM micrographs of the upper and side views of sugar-sheet formulations. In both plain and nine-pass (9)-printed films, the material structure presented a rough surface and an extended pore network. Similarities in the micrographs indicate the fact that the printing process did not affect the overall morphological characteristics of the sugar-sheet matrix. At this point, it is also important to mention that thermal inkjet printing deposits the API onto the substrate rather than dispersing it, so the printed films uphold their mechanical properties and their stability over time [23].

**Figure 3:**
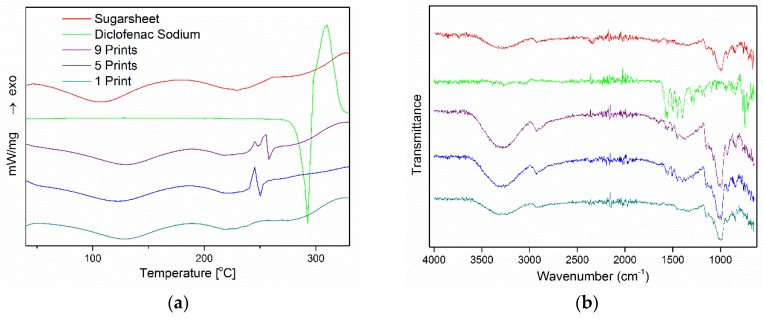
(**A**) DSC thermograms and (**B**) FTIR spectra of pure components and drug-loaded formulations.

The physicochemical properties of empty and drug-loaded films are illustrated in Figure 3. Figure 3A depicts the thermograms of plain materials (sugar sheet, DNa) and printed films under 1, 5, and 9 passes. A sharp endothermic peak, corresponding to the melting point, was observed at 295 °C for DNa, followed by an exothermic peak at 310 °C that implies decomposition of the API [24,25]. In accordance with the composition of substrates reported by the manufacturer and by previous studies [26,27], the plain sugar sheet presented two distinct broad endotherms in the temperature range of 50–250 °C, revealing the presence of bound water content and following the endothermic patterns of maltodextrin and sugar monosaccharides. Similar patterns were observed for printed formulations. In the case of one-pass-printed films, the absence of the DNa heat capacity minimum indicated that the drug content is either under the detection limit of the device or exists in a noncrystalline state. At 5- and 9-prints, the featured sharp endothermic peak of the API was present at lower temperatures (250 °C) with an inverted sequence of the exotherm, indicating partial molecular dispersion of DNa in the sugar-sheet matrix [28,29].

The FTIR spectra of the studied materials are illustrated in Figure 3B. Similar to thermal analysis, the distinct vibration of the substrate in the areas of 3000–3500 cm^−1^ and 1250–600 cm^−1^ is present in all formulations. DNa exhibits characteristic bands at 1572–1401 cm^−1^, which are associated with the symmetric and asymmetric stretching mode of the carboxylate group [24,30,31]. This vibration exists in both 5- and 9-printed films, indicating the total or partial presence of the API in a crystalline state. In the spectrum of the 1-printed film, no characteristic bands of DNa are observed, so it was hypothesized that the drug content is either under the detection limit of the device or molecularly dissolved in the substrate matrix.

**Figure 4:**
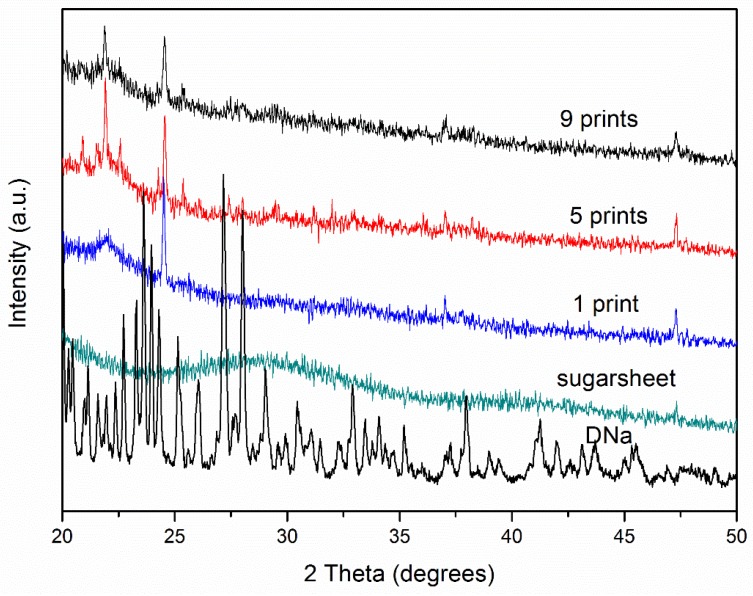
X-ray diffractograms of pure components and formulations.

The X-ray diffractograms of the raw materials and suggested formulations are shown in Figure 4. Characteristic sharp peaks at 23°, 24°, 26°, and 27° were observed in the pattern of DNa. Similar diffraction profiles were obtained from the formulated films. Herein, the solvation capacity of PG for DNa is key to the decreasing intensity under multiple prints. The more the layers were printed, the more PG was deposited onto the substrate and consequently absorbed into the matrix, maintaining a large amount of DNa in the amorphous state (considering the high solvation capacity of PG and evaporation of EtOH), while partial crystallinity of the API was observed. On the contrary, although the 1-printed film contains the minimum amount of drug, the diffractogram exhibits higher intensity and thus a higher fraction of API in a crystalline state, due to the minimum amount of PG as well.

**Figure 5:**
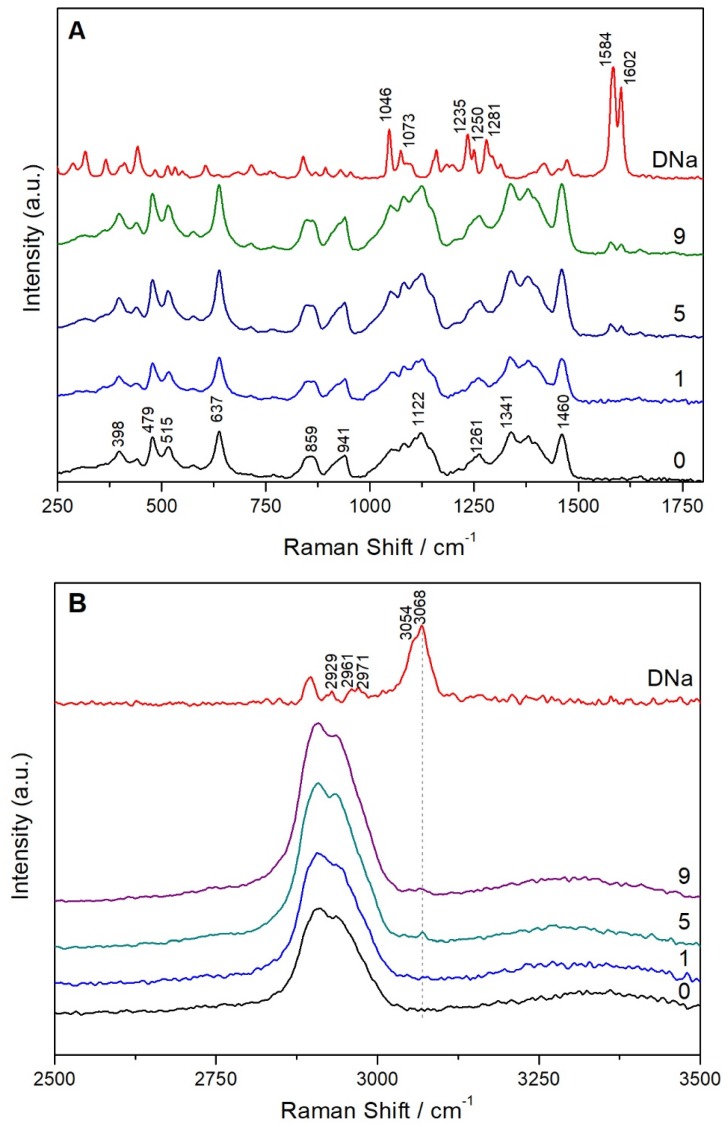
FT-Raman spectra in the range of 250–1800 cm^−1^ (**A**) and 2500–3500 cm^−1^ (**B**) of DNa; plain sugar sheet (0); and films printed with 1, 5, and 9 passes.

The FT-Raman spectra of pure DNa; sugar sheets; and films printed with 1, 5, and 9 passes are shown in Figure 5. DNa exhibits characteristic peaks at 1073 and 1046 cm^−1^, which are assigned to breathing vibrations of dichlorophenyl (ring 1) and phenylacetate (ring 2), respectively. The bands present at 1584 and 1602 cm^−1^ are attributed to stretching vibrations of ring 1 and ring 2, respectively. The bands located at 1235, 1250, and 1281 cm^−1^ are associated with rocking vibrations of the CH groups of both rings. The peaks at 2929, 2961, 2971, and 3054 cm^−1^ are due to CH stretching vibrations in ring 2, while the peak at 3068 cm^−1^ corresponds to the respective vibration in ring 1 [32]. The peaks at 1584, 1602, and 3068 cm^−1^ that can be clearly distinguished on the printed substrates indicated the presence of DNa. The sugar sheet is composed of many ingredients, especially sugars, polysaccharides, emulsifiers, and pigments. Furthermore, since sugars can exist in anhydrous or hydrated states or even form polymorphs, the interpretation of the corresponding FT-Raman spectrum is very complex.

### 3.5. In Vitro Release

**Figure 6:**
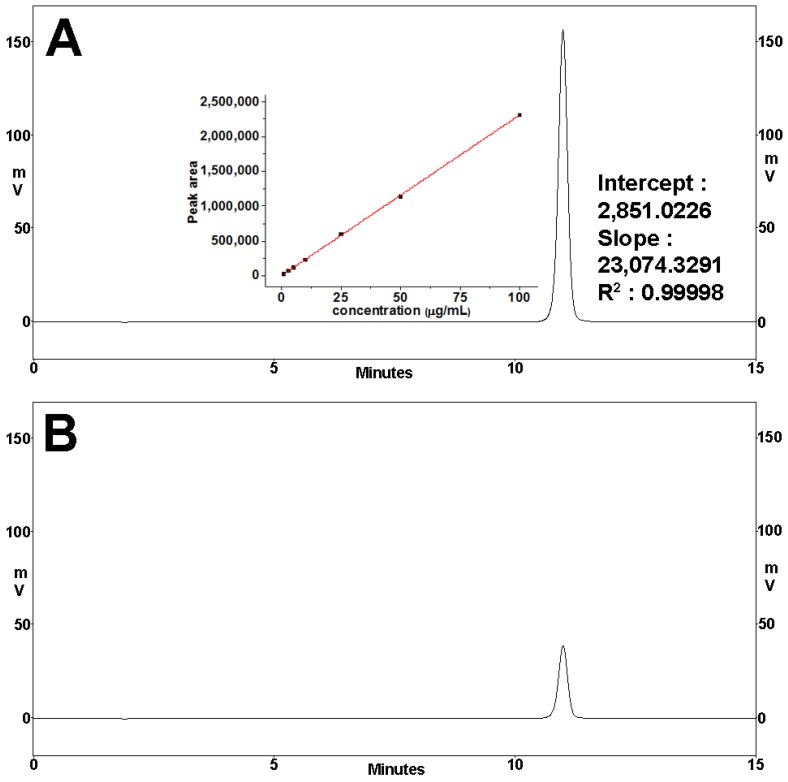
(**A**) HPLC spectrum of a representative diclofenac sodium calibration standard: concentration 100 μg/mL and fitting parameters of the respective calibration curve; (**B**) HPLC spectrum of a representative sample analysis during release experiments.

**Figure 7:**
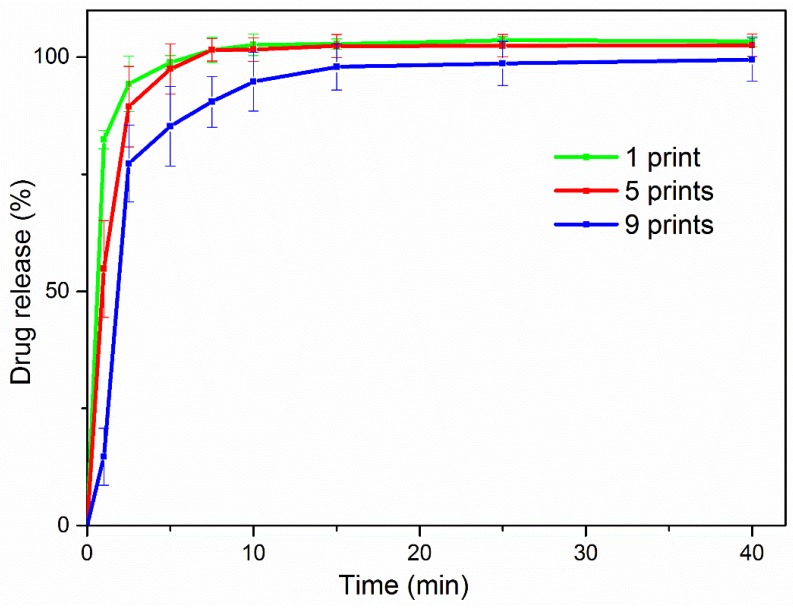
Release profiles of the developed formulations in simulated saliva.

Drug release was monitored in SS for 1 h. Figure 6 presents the typical HPLC spectra of DNa calibration standard analysis. Similar release profiles were obtained for both 1-, 5-, and 9-printed films, as depicted in Figure 7 (values are presented as mean ± SD, *n* = 3). All formulations released a large amount of the drug within 5 min: 85%, 97%, and 98%, in the order of increasing number of times the layer was printed. Almost all the amount of API was identified as being released within 10 min, and the profiles plateaued for the rest of the time period of the study. Negligible differences of the percentage of release values for each formulation in every time point were seen to be insignificant (*p* > 0.05). Assessment of fitting the implemented models is presented in Table 3. In all cases, the first-order kinetic model was optimally fit on the release profiles of the developed formulations, compared to the Korsmeyer–Peppas model, with R^2^ values in the range of 0.9714–0.9978 and 0.8108–0.9909, respectively.

### 3.6. Drug Permeation

**Figure 8:**
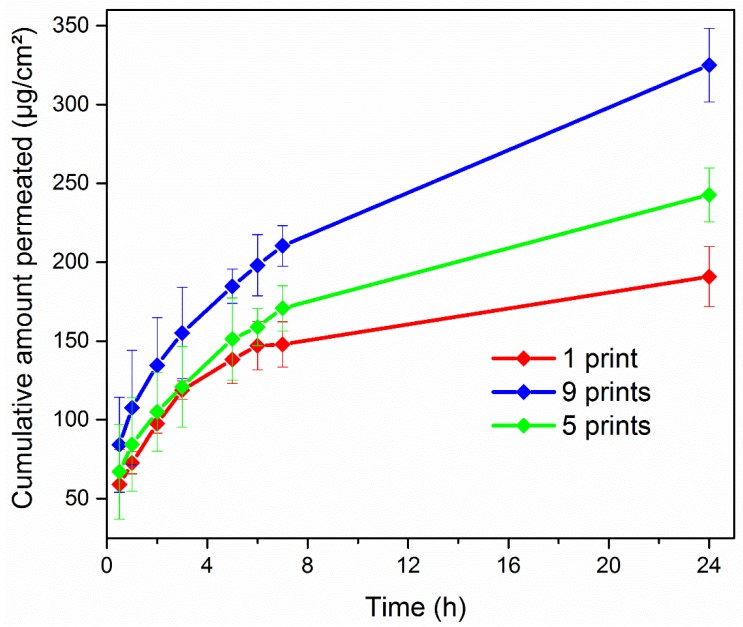
Cumulative transport of DNa across the cellulose membrane.

The in vitro permeation profiles of 1-, 5-, and 9-printed films are illustrated in Figure 8. In accordance with the drug load of each film, a significant increase of the cumulative amount that permeated the cellulose membrane was observed at each time point of the study, corresponding to the increasing printing-pass order of the films. In detail, the cumulative amount of the active transported through the membrane after 24 h from the 9-printed film was 1.5-fold and 2-fold greater than the measured values of the 5- and 1-printed films, respectively (*p* < 0.05). Table 4 shows the permeation parameters of the study. Variations of the drug content in the donor compartment resulted in different concentration gradients through the cellulose membrane. Thus, significant increases on the calculated steady-state flux and apparent permeability coefficient of each formulation (*p* < 0.05) were observed, with the latter presenting a gradual increase from 6.672 cm·h^−1^ to 12.386 cm·h^−1^ and finally 14.371 cm·h^−1^ for the 1-, 5-, and 9-printed films, respectively.

## 4. Conclusions

An edible buccal film (sugar sheet-based) containing the nonsteroidal anti-inflammatory drug diclofenac sodium has been developed by means of 2D printing technology. This technique offers a new and relatively safe way for handling potent drugs as well as creating sophisticated model delivery systems. Formulations made of multiple layers, coating materials, or APIs can be easily manufactured by 2D printing. Furthermore, substrates used in inkjet printing offer stability and adhesive properties enhancing the bioavailability of the drug [7].

Solubility studies showed that the greatest solubility of the API was achieved in mixtures of EtOH/PG, fulfilling at the same time the ideal rheological properties of liquid inks. Ethanol is evaporated after the printing procedure, and PG is a nontoxic, safe, and low-cost solvent [33] in which DNa is dissolved freely, offering the advantage of adjusting the dose and achieving very high or very low concentrations.

Diclofenac sodium is available on the market in certain doses. However, having the drug product predisposed to inkjet printing expands the variety of dosage forms, simultaneously improving the level of patient care in the context of personalized dosing [4]. Increasing doses of the active were produced by varying the number of substrate passes through the printing device. Physicochemical characterization of the films revealed that the API shifts to an amorphous state by increasing the number of passes through the printer.

A main concern in buccal delivery is the residence time of the formulation in the oral cavity, as saliva flow, chewing, swallowing, and speech may cause shearing in the oral cavity and obstruct the adhesion to the oral mucosa, resulting in low or no effectiveness to the patient. Burst release of the drug improves the kinetic profile of the API, as it is rapidly diffused from its carrier and can be easily absorbed from the mucus [34]. The in vitro release studies have shown a rapid release of the active within 10 min, whereas in vitro permeation studies revealed an increased apparent permeability versus the number of printed layers.

## Figures and Tables

**Table 1:** Solubility values of DNa investigated in various solvents.

Solvent	Solubility (mg/mL)
Distilled Water	33.7 ± 1.3
EtOH	74.3 ± 1.9
PEG	64.4 ± 1.8

**Table 2:** Viscosity values of the liquid inks.

EtOH:PG Ratio (% *v*/*v*)	DNa (mg·mL^−1^)	Kinematic Viscosity (mm^2^·s^−1^)	Density (g·cm^−3^)	Dynamic Viscosity (mPa·s)	Surface Tension (mN·m^−1^)
20:80	-	19.16 ± 0.03	0.923 ± 0.009	17.68 ± 0.04	-
40:60	-	9.08 ± 0.04	0.902 ± 0.010	8.19 ± 0.03	-
50:50	-	6.03 ± 0.07	0.868 ± 0.008	5.23 ± 0.03	25.7 ± 0.4
50:50	375.0	29.20 ± 0.05	0.982 ± 0.008	28.67 ± 0.04	-
50:50	227.3	18.29 ± 0.04	0.965 ± 0.007	17.64 ± 0.06	27.9 ± 0.5

**Table 3:** Curve fitting parameters.

Formulation	First Order Model		Korsmeyer–Peppas Model		
	k	R^2^	k	n	R^2^
1-printed	1.687	0.9952	88.20	0.0537	0.9909
5-printed	0.826	0.9978	74.26	0.1112	0.9364
9-printed	0.387	0.9714	52.08	0.2115	0.9108

**Table 4:** Permeation parameters for multiple printed films.

Printed Layers	J_ss_ (μg·cm^−2^·h^−1^)	P·10^−4^ (cm·h^−1^)
1	0.806 ± 0.014	6.672 ± 0.142
5	4.469 ± 0.193	12.386 ± 0.655
9	6.921 ± 0.248	14.371 ± 0.631

## References

[1] Alomari M., Mohamed F.H., Basit A.W., Gaisford S. (2015). Personalised dosing: Printing a dose of one’s own medicine. Int. J. Pharm..

[2] Magdassi S. (2010). The Chemistry of Inkjet Inks.

[3] Dudley J.T., Listgarten J., Stegle O., Brenner S.E., Parts L. (2014). Personalized medicine: From genotypes, molecular phenotypes and the quantified self, towrds improved medicine. Biocomputing 2015.

[4] Preis M., Breitkreutz J., Sandler N. (2015). Perspective: Concepts of printing technologies for oral film formulations. Int. J. Pharm..

[5] Kolakovic R., Viitala T., Ihalainen P., Genina N., Peltonen J., Sandler N. (2013). Printing technologies in fabrication of drug delivery systems. Expert Opin. Drug Deliv..

[6] Varan C., Wickström H., Sandler N., Aktaş Y., Bilensoy E. (2017). Inkjet printing of antiviral PCL nanoparticles and anticancer cyclodextrin inclusion complexes on bioadhesive film for cervical administration. Int. J. Pharm..

[7] Daly R., Harrington T.S., Martin G.D., Hutchings I.M. (2015). Inkjet printing for pharmaceutics—A review of research and manufacturing. Int. J. Pharm..

[8] Meléndez P.A., Kane K.M., Ashvar C.S., Albrecht M., Smith P.A. (2008). Thermal Inkjet Application in the Preparation of Oral Dosage Forms: Dispensing of Prednisolone Solutions and Polymorphic Characterization by Solid-State Spectroscopic Techniques. J. Pharm. Sci..

[9] Pardeike J., Strohmeier D.M., Schrödl N., Voura C., Gruber M., Khinast J.G., Zimmer A. (2011). Nanosuspensions as advanced printing ink for accurate dosing of poorly soluble drugs in personalized medicines. Int. J. Pharm..

[10] Scoutaris N., Alexander M.R., Gellert P.R., Roberts C.J. (2011). Inkjet printing as a novel medicine formulation technique. J. Control. Release.

[11] Buanz A.B.M., Saunders M.H., Basit A.W., Gaisford S. (2011). Preparation of Personalized-dose Salbutamol Sulphate Oral Films with Thermal Ink-Jet Printing. Pharm. Res..

[12] Genina N., Fors D., Palo M., Peltonen J., Sandler N. (2013). Behavior of printable formulations of loperamide and caffeine on different substrates—Effect of print density in inkjet printing. Int. J. Pharm..

[13] Genina N., Janßen E.M., Breitenbach A., Breitkreutz J., Sandler N. (2013). Evaluation of different substrates for inkjet printing of rasagiline mesylate. Eur. J. Pharm. Biopharm..

[14] Vakili H., Wickström H., Desai D., Preis M., Sandler N. (2017). Application of a handheld NIR spectrometer in prediction of drug content in inkjet printed orodispersible formulations containing prednisolone and levothyroxine. Int. J. Pharm..

[15] Wickström H., Broos A., Nyman J.O., Kortesmäki E., Eklund P., de Beer T., Preis M., Sandler N. (2018). Handheld colorimeter as quality control tool for inkjet printed flexible levothyroxine doses for pediatric use. Int. J. Pharm..

[16] Kollamaram G., Hopkins S.C., Glowacki B.A., Croker D.M., Walker G.M. (2018). Inkjet printing of paracetamol and indomethacin using electromagnetic technology: Rheological compatibility and polymorphic selectivity. Eur. J. Pharm. Sci..

[17] Vuddanda P.R., Alomari M., Dodoo C.C., Trenfield S.J., Velaga S., Basit A.W., Gaisford S. (2018). Personalisation of warfarin therapy using thermal ink-jet printing. Eur. J. Pharm. Sci..

[18] Edinger M., Bar-Shalom D., Sandler N., Rantanen J., Genina N. (2018). QR encoded smart oral dosage forms by inkjet printing. Int. J. Pharm..

[19] Chuasuwan B., Binjesoh V., Polli J.E., Zhang H., Amidon G.L., Junginger H.E., Midha K.K., Shah V.P., Stavchansky S., Dressman J.B. (2009). Biowaiver Monographs for Immediate Release Solid Oral Dosage Forms: Diclofenac Sodium and Diclofenac Potassium. J. Pharm. Sci..

[20] Calixto G., Garcia M., Cilli E., Chiavacci L., Chorilli M. (2016). Design and Characterization of a Novel p1025 Peptide-Loaded Liquid Crystalline System for the Treatment of Dental Caries. Molecules.

[21] Khan S., Boateng J. (2018). Effects of Cyclodextrins (β and γ) and l-Arginine on Stability and Functional Properties of Mucoadhesive Buccal Films Loaded with Omeprazole for Pediatric Patients. Polymers.

[22] Marques M.R.C., Loebenberg R., Almukainzi M. (2011). Simulated Biological Fluids with Possible Application in Dissolution Testing. Dissolut. Technol..

[23] Buanz A.B.M., Belaunde C.C., Soutari N., Tuleu C., Gul M.O., Gaisford S. (2015). Ink-jet printing versus solvent casting to prepare oral films: Effect on mechanical properties and physical stability. Int. J. Pharm..

[24] Yazdi A.K., Smyth H.D.C. (2016). Hollow crystalline straws of diclofenac for high-dose and carrier-free dry powder inhaler formulations. Int. J. Pharm..

[25] Balogh A., Horváthová T., Fülöp Z., Loftsson T., Harasztos A.H., Marosi G., Nagy Z.K. (2015). Electroblowing and electrospinning of fibrous diclofenac sodium-cyclodextrin complex-based reconstitution injection. J. Drug Deliv. Sci. Technol..

[26] Elnaggar Y.S.R., El-Massik M.A., Abdallah O.Y., Ebian A.E.R. (2010). Maltodextrin: A Novel Excipient Used in Sugar-Based Orally Disintegrating Tablets and Phase Transition Process. AAPS PharmSciTech.

[27] Ruiz-Cabrera M.A., Schmidt S.J. (2015). Determination of glass transition temperatures during cooling and heating of low-moisture amorphous sugar mixtures. J. Food Eng..

[28] AL-Kahtani A.A., Sherigara B.S. (2014). Controlled release of diclofenac sodium through acrylamide grafted hydroxyethyl cellulose and sodium alginate. Carbohydr. Polym..

[29] Barzegar-Jalali M., Alaei-Beirami M., Javadzadeh Y., Mohammadi G., Hamidi A., Andalib S., Adibkia K. (2012). Comparison of physicochemical characteristics and drug release of diclofenac sodium-eudragit^®^ RS100 nanoparticles and solid dispersions. Powder Technol..

[30] Bukara K., Drvenica I., Ilić V., Stančić A., Mišić D., Vasić B., Gajić R., Vučetić D., Kiekens F., Bugarski B. (2016). Comparative studies on osmosis based encapsulation of sodium diclofenac in porcine and outdated human erythrocyte ghosts. J. Biotechnol..

[31] Gaitano R.O., Calvo N.L., Narda G.E., Kaufman T.S., Maggio R.M., Brusau E.V. (2016). Preparation and Physical Characterization of a Diclofenac-Ranitidine Co-precipitate for Improving the Dissolution of Diclofenac. J. Pharm. Sci..

[32] Iliescu T., Baia M., Miclăuş V. (2004). A Raman spectroscopic study of the diclofenac sodium–β-cyclodextrin interaction. Eur. J. Pharm. Sci..

[33] Shayanfar A., Acree W.E., Jouyban A. (2009). Solubility of Lamotrigine, Diazepam, Clonazepam, and Phenobarbital in Propylene Glycol + Water Mixtures at 298.15 K. J. Chem. Eng. Data.

[34] Fonseca-Santos B., Chorilli M. (2018). An overview of polymeric dosage forms in buccal drug delivery: State of art, design of formulations and their in vivo performance evaluation. Mater. Sci. Eng. C.

